# Supplementary material for: Identification of photoperiod-induced specific miRNAs in the adrenal glands of Sunite sheep (Ovis aries)
Source: Front Vet Sci. 2022 Jul 22;9:888207. doi: 10.3389/fvets.2022.888207 (PMC9354845; doi:10.3389/fvets.2022.888207)
Supplement: Supplementary file 1 [file Data_Sheet_1.ZIP › Supplementary file/miRDeep2 file.zip!/miRDeep2 file/Know_Structure/SPLP42A3/oar-mir-323a.pdf]

miRBase precursor : oar-mir-323a
Total read count : 1484
oar-miR-323a-5p read count : 12
oar-miR-323a-3p read count : 1472
remaining reads : 0

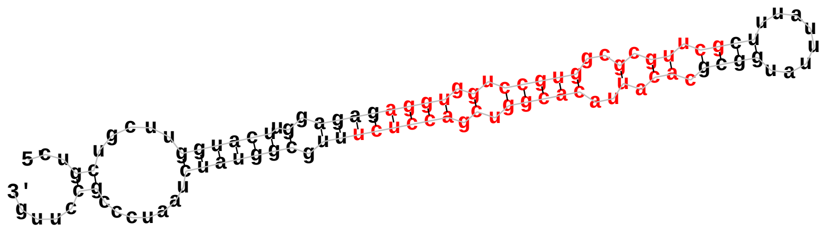

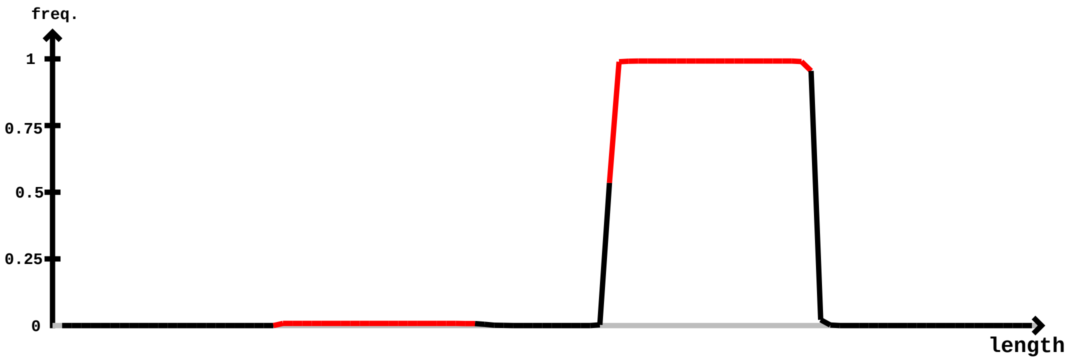


```
                                         oar-miR-323a-5p
                                                                   oar-miR-323a-3p
5'- cugcugcuugguacuuggagagagguggucc guggcgcguucgcuuuauuuauggcgcacauuacacggucgaccucuuugcgguaucuaaucccgccuug  -3'   exp
    .........((((((.(.((((((((.(.(((((..(.((.((((........)))).)).)..))))).).)))))))).))))))).............  reads   mm   sample
    ......................agguggucc guggcgcguu............................................................  1       0    seq
    ......................agguggucc guggcgcguucg..........................................................  4       0    seq
    ......................agguggucc guggcgcguucgc.........................................................  5       0    seq
    ......................agguggucc guggcgcguucgcu........................................................  1       0    seq
    ......................agguggucc guggcgcguucgcuu.......................................................  1       0    seq
    .......................................................cgcacauuacacggucgaccucu.......................  4       0    seq
    ........................................................gcacauuacacggucgaccu.........................  2       0    seq
    ........................................................gcacauuacacggucgaccuc........................  26      0    seq
    ........................................................gcacauuacacggucgaccucu.......................  753     0    seq
    ........................................................gcacauuacacggucgaccucuu......................  9       0    seq
    .........................................................cacauuacacggucgaccu.........................  1       0    seq
    .........................................................cacauuacacggucgaccuc........................  26      0    seq
    .........................................................cacauuacacggucgaccucu.......................  624     0    seq
    .........................................................cacauuacacggucgaccucuu......................  20      0    seq
    .........................................................cacauuacacggucgaccucuuu.....................  3       0    seq
    ..........................................................acauuacacggucgaccucu.......................  3       0    seq
    ...........................................................cauuacacggucgaccucu.......................  1       0    seq
```
